# Sacral Neuromodulation: Foray into Chronic Pelvic Pain in End Stage Endometriosis

**DOI:** 10.1155/2017/2197831

**Published:** 2017-03-06

**Authors:** Maija Lavonius, Pia Suvitie, Pirita Varpe, Heikki Huhtinen

**Affiliations:** Department of Digestive Surgery and Department of Gynecology and Obstetrics, Turku University Hospital, Turku, Finland

## Abstract

Excision of all endometriotic lesions is the method of choice in the treatment of severe endometriosis resistant to medical therapy. The infiltrating nature of the disease as well as extensive surgery may, however, cause chronic pain that cannot be relieved by either surgery or hormonal treatment. As a pilot treatment, we tested the effect of sacral neuromodulation (SNM) for four endometriosis patients suffering chronic pelvic pain and pelvic organ dysfunction after radical surgical treatment. Three out of four patients reported improvement in their symptoms during the neuromodulation testing period and a permanent pulse generator was installed. After 2.5 years, all three patients report better quality of life and want to continue with SNM.

## 1. Introduction

Sacral neuromodulation (SNM) involves electrical modulation of a sacral nerve root by means of an electrode and a pulse generator. The conventional indications for SNM are urinary retention, urinary incontinence, anal incontinence, and constipation [1]. Several studies have also demonstrated promising results of SNM in chronic pelvic pain [2], although its mechanism of action remains ill defined [3]. One study has evaluated the effect of SNM on voiding dysfunction after surgery for deep infiltrating (DIE) endometriosis [4]. To our knowledge, no reports of SNM for the treatment of pelvic pain after radical surgery of endometriosis have been published.

Endometriosis is an estrogen-dependent chronic inflammatory disease defined by the presence of functional endometrial tissue outside the uterine cavity. Approximately 4–10% of women are affected during their fertile years suffering from a variety of pelvic pain symptoms and subfertility [5]. Endometriosis significantly reduces health-related quality of life (HRQoL) mostly due to pain symptoms [6]. Pain can be alleviated with hormonal medication and nonsteroidal anti-inflammatory drugs (NSAID) [7]. When medical therapy fails, complete surgical excision of the endometriotic lesions is the method of choice. In severe DIE, a multidisciplinary approach can be necessary.

Endometriosis can cause pain in many different mechanisms [8]. Irritation of pelvic nerves due to inflammation causes nociceptive pain. In addition, DIE can infiltrate pelvic nerves causing pain, and de novo nerve growth is also detected in ectopic endometrial tissue. During surgery, all efforts are made to preserve the pelvic nerves. However, in severe cases, nerve-sparing technique is not possible if the goal is to remove all endometriosis. In such cases, a compromise is made between radicalness of surgery and preservation of pelvic organ function. The disease itself as well as extensive surgery can lead to chronic neuropathic pain. In such cases, little options are available to ease the pain.

## 2. Case Presentation

Common to all our four patients was a history of surgically treated deep infiltrating endometriosis in lateral pelvic sidewalls affecting ureters, sacrouterine ligaments, and pelvic nerves. All women had undergone hysterectomy and bilateral salpingo-oophorectomy combined with excision of all macroscopic endometriosis. Thus, adenomyosis as the source of pain was excluded. No recurrent endometriosis was suspected in gynecological examination and transvaginal ultrasound, and the chronic noncyclical pelvic pain was life interfering and resistant to hormonal and medical treatment. Detailed surgical history is presented in Table 1. All women were fully informed of the experimental nature of the SNM treatment for this indication. No ethics approval was applied because of acknowledged use of SNM for other indications with pelvic disorders. SNM was offered to one more patient but she declined because she feared the procedure.

The SNM procedure with InterStim II™ system was performed in two stages. In the first stage, a permanent lead was implanted under local anesthesia with fluoroscopic control into S3 or S4 sacral foramina as described in detail earlier [3]. In our patients, the permanent lead was implanted into the foramen with the best motor and sensory response in the testing phase (Table 1). In the second stage, permanent pulse generator was implanted for the patients with significant objective relief of symptoms after 3-4 weeks testing period with external pulse generator. In those with negative symptom relief, the lead was removed under local anesthesia.

A self-designed disease-related questionnaire was used to assess pelvic pain symptoms as well as bowel and urogenital function before and 3–6 months after SNM procedure. This questionnaire evaluated the presence of abdominal or pelvic pain or dyspareunia; functional bowel symptoms like pain and difficulties on defecation, fecal incontinence, or constipation; urinary symptoms like incomplete bladder emptying, dysuria, and urinary incontinence. In addition, patients were advised to keep daily pain diary and pain medication diary for fourteen days before, during, and after SNM test period. Furthermore, women with permanent pulse generator were asked to evaluate the effect of SNM treatment to pelvic pain symptoms and bowel and bladder function 3–6 months after procedure (1: worse, 2: no change, 3: somewhat improved, 4: much improved, 5: excellent improvement). Women's subjective satisfaction on SNM was evaluated with numerical rating scale (NRS) (0 meaning totally unsatisfied and 10 meaning totally satisfied). During the treatment and follow-up, women had the possibility for daily contact with study nurse.

Main pre-SNM symptoms and self-reported response to treatment are shown in Table 2.

After the test period, three patients reported considerable or excellent improvement (NRS 8-9) in subjective quality of life and were offered to have permanent pulse generator installed. Two women found considerable improvement in bladder symptoms and two women found considerable or excellent relief in dyspareunia. All these three women found that SNM relieved chronic pelvic pain a little, but the pain was easier to tolerate when functional pelvic symptoms eased. Total use of pain medication decreased, but only one woman filled the questionnaire fully adequately regarding NRS values and pain medication.

Patient number four did not experience any improvement in symptoms during the test period. Permanent pulse generator was not installed. She did not return any of the questionnaires.

We organized a phone call control to all four patients 2.5 years after the test period. All three patients with SNM installed wanted to continue with SNM. None of the patients with SNM were totally symptom-free but they all reported better quality of life by better symptom control enabling working, social life, and travelling. Main pre-SNM symptom for patient 1 was disabling chronic pain and defecation problems. She got excellent help for pain and dyspareunia from SNM but the first modulator had to be removed because of infection. Second modulator gave good pain control but it has not been as good with dyspareunia. For good symptom control, she has needed SNM reprogramming 2-3 times a year. Patient 2 announced that chronic pelvic pain and pain during defecation had eased as well as fecal and urinary incontinence. Patient 3 had excellent help for obstipation and lived a regular life. She was fully satisfied with SNM. Fourth patient has retired in the age of 38 and suffers chronic pelvic pain, functional bowel symptoms, dysuria, and dyspareunia. No spontaneous symptom mitigation has occurred during 2.5 years. She underwent full clinical and radiological reevaluation with MRI year 2015 and has tried all hormonal treatments and pain killers without help.

## 3. Discussion

Our clinical experience is that deep endometriotic nodules at area of uterosacral ligaments with infiltration to hypogastric nerves and the ureters create the most difficult disease to treat. Possibilities of surgery are limited by preservation of vascular, urinary, and neural structures. Nature of the pain is often neurogenic and response to hormonal treatment and regular pain medication is poor. Luckily, this end stage is rare but those few patients may end up being quite desperate.

SNM has been used for pelvic floor pain and functional pelvic disorders for other indications but not for endometriosis [9]. We decided to propose SNM on experimental basis for women who had tried all other possible treatments.

A self-administered disease-related questionnaire was used to assess problems with bowel and urogenital functions, but the compliance was poor. After the SNM test period, three out of four patients announced significant relief in their symptoms and a permanent pulse generator was implanted. NRS did not turn out to be reliable indicator of pain since the heavy use of pain medication in the beginning of study biased the results. However, three out of four patients were able to reduce pain medication. These three women also felt that problems with defecation and urinary symptoms were alleviated significantly. In addition, two women got a remarkable help for dyspareunia and reported great increase in subjective quality of life. All three patients want to continue with the SNM after 2.5 years. Fourth patient who did not benefit from SNM has not experienced any spontaneous relief in symptoms during 2.5 years.

We are fully aware of the placebo effect of SNM. However, since three out of four patients with chronic pelvic pain and pelvic floor dysfunction reported a clinically plausible benefit of this pilot trial, we believe that SNM for end stage endometriosis patients is worth further controlled studies.

## Figures and Tables

**Table 1:** Surgical history of the four women in the neuromodulation pilot study. Operations were performed by laparotomy if laparoscopy is not mentioned in the text.

Patient	Age (years)	year	Operation	Indication	Postoperative complications
1 G2P2		1999	Laparoscopy	Endometriosis	None
		2000	Laparoscopic left salpingo-oophorectomy	Endometriosis	None
		2000	Laparoscopic right salpingo-oophorectomy, supracervical hysterectomy	Endometriosis	None
		2009	Adhesiolysis and extirpation of the uterine cervix	Pelvic pain	None
		2011	Anterior resection, appendicectomy, and adhesiolysis	Pelvic pain (left side)	None
	**43**	2014	Neuromodulation test period and a permanent pulse generator installation (right S4)	Pelvic pain	None
2 G0P0		2002	Left salpingo-oophorectomy and adhesiolysis	Endometriosis	None
		2006	Anterior resection and ileostomy	Sigmoid perforation	Rectal anastomosis stricture
		2007	Reanterior resection and ileostomy closure		None
		2009	Adhesiolysis, ileum resection, and supracervical hysterectomy	Postoperative adhesions, bowel obstruction	None
		2013	Re-reanterior resection, resection of left ureter, extirpation of the uterine cervix, and ileostomy	Endometriosis (main symptom pain) and anal incontinence and defecation difficulties	Ureteral anastomosis leakage treated by pyelostomy and drainage
		2013	Ileostomy closure		None
	**50**	2014	Neuromodulation test period and a permanent pulse generator installation (left S4)	Pelvic pain and defecation difficulties	None
3 G2P2		1991	Laparoscopy	Endometriosis	None
		1995	Laparoscopy	Endometriosis	None
		2003	Laparoscopic excision of sacrouterine ligaments and electrocoagulation of peritoneal endometriosis	Endometriosis	None
		2005	Laparoscopic electrocoagulation of peritoneal endometriosis and adhesiolysis	Endometriosis	None
		2008	Hysterectomy and excision of peritoneal endometriosis	Endometriosis	None
		2010	Laparoscopic adhesiolysis and left salpingo-oophorectomy	Postoperative adhesions, pain, left ovarian cyst	None
		2010	Vaginal resection, extirpation of left periureteral endometriosis	Endometriosis, hydronephrosis	Laparotomy wound infection
	**42**	2014	Neuromodulation testing period and a permanent pulse generator installation (right S4)	Pelvic pain and defecation difficulties	None
4 G1P0		2011	Hysterectomy and bilateral salpingectomy	Endometriosis	None
		2012	Ileum resection, appendicectomy, bilateral oophorectomy, vaginal resection, and resection of left sacrouterine ligament	Endometriosis, pelvic pain	None
	**35**	2014	Neuromodulation testing period (left S3)	Pelvic pain	None

*Note*. G: gravidity, P: parity.

**Table 2:** Main pre-SNM symptoms and response to SNM treatment.

Patient	1			2			3		
Time point to SNM	Pre	0.5 yrs	2.5 yrs	Pre	0.5 yrs	2.5 yrs	Pre	0.5 yrs	2.5 yrs
*Symptom*									
Abdominal or pelvic pain	yes	4	5	yes	4	4	yes	3	4
Dyspareunia	yes	4	3	yes	4	3	yes	na	na
Dyschezia or bowel colic	yes	5	4	yes	4	4	no	na	na
Constipation, outlet obstruction	yes	5	3	no	na	na	yes	4	5
Anal incontinence	yes	5	4	yes	4	3	no	na	na
Dysuria	no	na	na	yes	4	5	no	na	na
Voiding dysfunction	yes	4	2	no	na	na	no	na	na
Urinary incontinence	yes	4	2	yes	4	4	no	na	na
Satisfaction to SNM (NRS)		8	8		9	9		9	10

Compared to pre-SNM status:

1: worse, 2: no change, 3: somewhat improved, 4: much improved, 5: excellent improvement.

NRS: numerical rating scale 0–10.

na: not applicable.

## Abbreviations

SNM: Sacral neuromodulation
NRS: Numerical rating scale
DIE: Deep infiltrating endometriosis
NSAID: Nonsteroidal anti-inflammatory drug.

## Additional Points

*Précis.* Sacral neuromodulation may relieve pelvic pain and functional pelvic disorders in endometriosis patients resistant to surgical and medical treatment.
